# Constructing high-quality 1D nano/microwire hybrid structure for high-performance photodetectors based on CdSe nanobelt/perovskite microwire

**DOI:** 10.1515/nanoph-2023-0106

**Published:** 2023-02-28

**Authors:** Li Ren, Qiuhong Tan, Kunpeng Gao, Peizhi Yang, Qianjin Wang, Yingkai Liu

**Affiliations:** College of Physics and Electronic Information, Yunnan Normal University, Yunnan Kunming 650500, China; Yunnan Provincial Key Laboratory for Photoelectric Information Technology, Yunnan Normal University, Yunnan Kunming 650500, China; Key Laboratory of Advanced Technique & Preparation for Renewable Energy Materials, Ministry of Education, Yunnan Normal University, Kunming 650500, China

**Keywords:** all-inorganic perovskite, CdSe nanobelt, hybrid device, photoelectric characteristics

## Abstract

All-inorganic perovskite CsPbBr_3_ is considered as a promising photoelectric material due to its high environmental stability and excellent photoelectric properties. Constructing low-dimension hybrid structures by combining CsPbBr_3_ with semiconductor materials have recently attracted particular attention because they may bring new functionalities or generate synergistic effects in optoelectronic devices. Herein, the high-quality 1D CdSe nanobelt (NB)/CsPbBr_3_ microwire (MW) photodetectors are designed first time, which exhibit excellent performance as integrating *I*_on_/*I*_off_ ratio of 5.02 × 10^4^, responsivity of 1.63 × 10^3^ A/W, external quantum efficiency of 3.8 × 10^5^% and detectivity up to 5.33 × 10^12^ Jones. These properties are all improved at least one order of magnitude compared to those of single CsPbBr_3_ photodetectors. Moreover, the response range is broadened from the 300–570 nm (the single CsPbBr_3_ device) to 300–740 nm (the hybrid photodetector). Then, the first-principles calculations are carried out to reveal the physical mechanism from the atomic scale. The remarkably improved optoelectronic properties are attributed to the high crystalline quality as well as unique band alignment of hybrid structure that facilitate the effective separation and transport of photogenerated carriers. These works indicate that 1D CdSe/CsPbBr_3_ hybrid devices have promising applications in building high-performance and broader spectral response photodetectors and other optoelectronic devices.

## 1 Introduction

In recent decades, halide perovskite materials have been widely studied in optoelectronic devices due to their excellent photoelectric performance, including high absorption coefficient, low exciton binding energy and long carrier diffusion length, etc. [1]. To date, different shapes of perovskite photodetectors have been widely developed, such as thin film, bulk single crystal, and micro/nanowire photodetectors [2–12]. These photodetectors have made great strides in improving their performance and can outperform traditional semiconductor devices. Due to the inherent internal instability of the A-site organic cation, organic–inorganic hybrid perovskite is particularly unstable under moisture, oxygen, light, and high temperatures [13–15]. In contrast, the formation of all-inorganic perovskite by replacing organic cations with inorganic cations at the A-site not only overcomes the stability problem, but also does not deteriorate the transport performance, and thus maintains the efficiency of device [16]. This makes all-inorganic perovskite materials become an important supplement and substitute for organic–inorganic hybrid perovskite materials in new optoelectronic devices [17–19].

All-inorganic perovskite CsPbBr_3_ is a direct bandgap semiconductor, which possesses low trap state density, high carrier mobility, long electron hole diffusion length, high quantum yield, strong light absorption, high luminous efficiency, and other excellent optical properties [20]. Currently, the photodetector devices based on CsPbBr_3_ have been widely reported [21, 22]. However, the worse performance of all-inorganic perovskite photodetectors compared to that of organic–inorganic hybrid perovskites hinders their practical application and forces researchers to develop more ways to improve the performance of all-inorganic perovskite photodetectors. By combining perovskite with other functional materials to prepare composite structure has proved to be an effective approach for improving the device performance. Recently, the photodetector based on CsPbBr_3_ perovskite integrated with two-dimensional materials have been fabricated and the improved performance has been reported [23–28]. However, only partial key parameters of photodetector are improved, and more key parameters simultaneously improved are challenging as well as necessary for commercial application of the device. Therefore, more efforts are urgently needed to further improve the performance of CsPbBr_3_-based photodetector. 1D semiconducting micro/nano structures, which have fewer grain boundaries and lower defect density as well as their unique electrical and optical properties, are considered as a promising candidate as fundamental building blocks for optoelectronic device [29–32]. Inspired by this, fabricate 1D/1D hybrid structure is expected to provide a new route for improving the performance of photodetector. CdSe NBs have attracted increasing attention due to their excellent carrier mobility with a bandgap of 1.74 eV [33], and is an ideal perfect material combined with 1D CsPbBr_3_ to fabricate 1D/1D hybrid structure photodetectors. So far, the CdSe NB/CsPbBr_3_ MW 1D hybrid structures have not been reported yet.

In this work, the high-performance photodetector based on CdSe NB/CsPbBr_3_ MW 1D hybrid structures were constructed for the first time through the solution method combined with chemical vapor deposition (CVD) method. The results show that the CdSe NB/CsPbBr_3_ MW 1D hybrid structure photodetector not only has an increased spectral response range but also has a significantly improved performance compared to the single CsPbBr_3_ photodetector. The first-principles calculations indicated that the large interface potential barrier as well as band offset and interface band gap states are responsible for the improved performance of the hybrid structure.

## 2 Experimental section

### 2.1 Materials

Xi’an Bao Laite Optoelectronics Technology Co., Ltd. provided CsBr powders with a purity of 99.99 percent and PbBr_2_ powders with a purity of 99.99 percent. Tianjin Zhiyuan Chemical Reagent Co., Ltd. provided the dimethyl sulfoxide (DMSO: anhydrous solvent grade). Dow Corning provided the PDMS prepolymers and curing chemicals. All of the goods listed above were utilized without further purification.

### 2.2 Preparation of CsPbBr_3_ and CdSe NBs

CsPbBr_3_ MWs were prepared by in-plane self-assembly method. Firstly, equimolar CsBr and PbBr_2_ were dissolved in DMSO solution and stirred in an oil bath at 65 °C for 6 h. Then it was cooled to room temperature, filtered through a 0.45 m needle filter, and then pipette 10 μL drops of the precursor solution onto the cleaned silica substrate (vacuum plasma treatment for 10 min before use). Secondly, the precursor solution was covered with smooth and flat PDMS template and dried overnight on a 45 °C hot table. Finally, CsPbBr_3_ MWs were obtained after removing the covered PDMS template, the preparation flow chart is shown in Figure S1 (Supporting Information). CdSe NBs was synthesized by the experimental method reported by our previous works [2].

### 2.3 Preparation of CdSe/CsPbBr_3_ photodetectors

Firstly, the CsPbBr_3_ MWs were grown on the Si substrate. Then, the CdSe dispersion (dispersed in ethanol solution) was aspirated using a pipette dropped on the CsPbBr_3_ MWs substrate and dried. Finally, the Au (80 nm) electrode was evaporated on the CdSe NB/CsPbBr_3_ MW hybrid material by the electron beam evaporation system, and the CdSe NB/CsPbBr_3_ MW hybrid device was prepared. The process flow of CdSe NB/CsPbBr_3_ MW device is shown in Figure S2 (Supporting Information).

### 2.4 Theoretical calculation method

All the calculations were carried out within the framework of the DFT-LCAO method as implemented in QuantumATK S-2021.06-SP2 software package. The convergence criterion for the total energy and force were 10^−5^ eV and 0.005 eV/Å per atom, respectively. The cut off energy was set to 105 Hartree in all our calculations. The optimized constants are *a* = 8.3 Å, *b* = 8.55 Å and *c* = 11.99 Å for CsPbBr_3_ and *a* = *b* = 4.43 Å and *c* = 7.21 Å for CdSe, respectively. The lattice mismatch for the heterostructure are about 1.82% and 3.89% in *a* and *b* axial directions, respectively. A 3 × 4 × 79 *k*-point grid was adopted for the device model. The length of the center region of the device model is about 106.8 Å, which consists of 204 atoms. The HSE hybrid functional was used for calculating the band alignment of the heterostructure more accurately. The periodic, periodic and Dirichlet boundary conditions are used in the *x*, *y*, and *z* directions, respectively.

## 3 Results and discussion

**Figure 1:**
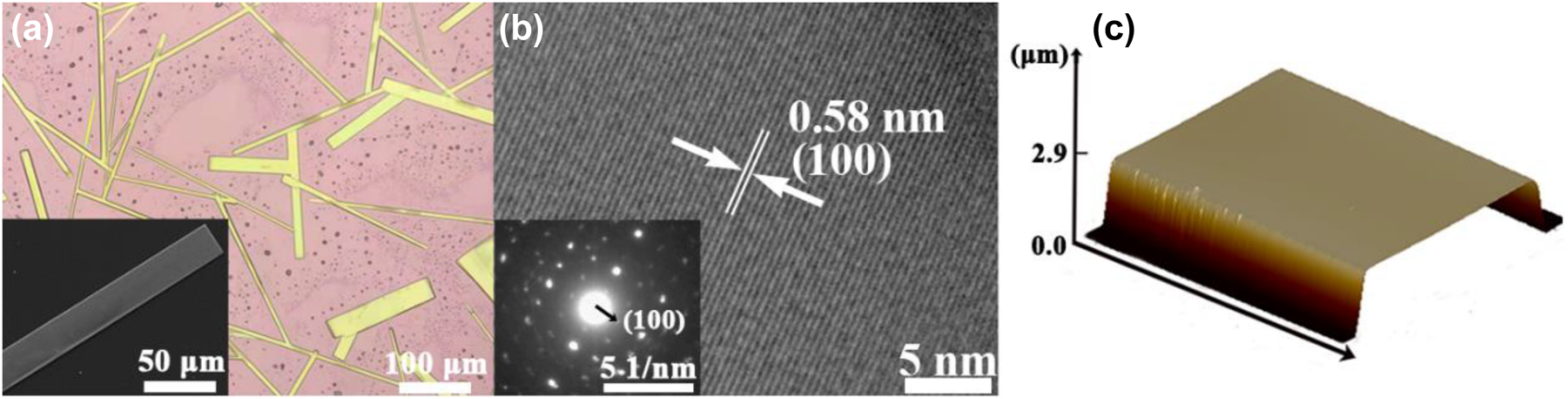
Microstructure images of CsPbBr_3_ MWs. (a) Optical microscopy image of CsPbBr_3_ MWs. Inset: SEM of single CsPbBr_3_ MW. (b) HRTEM image of CsPbBr_3_ MW. Inset: SAED pattern. (c) 3D AFM diagrams of single CsPbBr_3_ MW.

Figure 1(a) is optical images of the fabricated CsPbBr_3_ MWs, which show regular long line shapes with different widths. Figure S3a (Supporting Information) shows the energy dispersive X-ray spectroscopy (EDS) of single CsPbBr_3_ MW. It can be seen that the atomic ratios of Cs, Pb, and Br are about 1:1:3, which is corresponding to the stoichiometric ratio of CsPbBr_3_. Figure S3b–f (Supporting Information) displays a scanning electron microscope (SEM) image of a single CsPbBr_3_ MW and its corresponding Cs, Pb, and Br elements mapping. It is clearly seen that the CsPbBr_3_ MW have a smooth surface and the uniform element distribution, indicating that CsPbBr_3_ crystal has good crystallization quality and fewer defects, which is beneficial to improve the stability and optoelectronic properties of CsPbBr_3_ MW [34]. The high-resolution transmission electron microscopy (HRTEM) image and selected area electron diffraction (SAED) pattern of CsPbBr_3_ MW as presented in Figure 1(b). The clear lattice fringes with an interplane distance of 0.58 nm is corresponding to the (100) plane of CsPbBr_3,_ which indicated that the prepared CsPbBr_3_ MW is single crystal. Three-dimensional atomic force microscopy (3D AFM) in Figure 1(c) shows that the thickness of CsPbBr_3_ MW is about 2.9 μm. The 3D AFM image also demonstrated that the CsPbBr_3_ MW has a super-smooth and flat surface, which is conducive to maintain good contact with CdSe NB and resulting in the separation and transfer process of photogenerated carriers more unobstructed between them.

**Figure 2:**
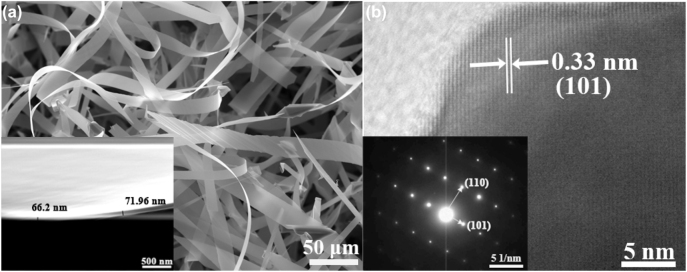
Microstructure images of CdSe NBs. (a) SEM and thickness (inset) and (b) HRTEM images and SAED patterns (inset) of CdSe NBs.

Figure 2(a) is the SEM image of prepared CdSe NBs by CVD method. It can be seen that each CdSe NB is uniform in width as well as thickness, and their surfaces and edges are smooth. The enlarged image of a single CdSe NB in Figure 2(a) inset indicated that the thickness of prepared CdSe NB is ∼70 nm. The HRTEM image shows clear lattice fringes, and the spacing between adjacent crystal planes is 0.33 nm, which corresponding to (101) crystal planes of CdSe. The crystal quality of prepared CdSe NBs was further characterized by SAED patterns, as shown in Figure 2(b) inset. The clear and regular diffraction spots in the SAED map further indicate that the grown CdSe NBs are high quality single-crystalline.

Figure S4 shows the EDS of CdSe NBs dispersed on the silicon substrate. It is obviously that the atomic ratio of Cd and Se is equivalent to the stoichiometric ratio of CdSe. The appearance of silicon peak demonstrated that the prepared CdSe NBs are quite thin, which is consistent with the previous SEM result in Figure 2(a). The EDS mapping shows that the spatially uniform elemental distribution of Cd and Se in the dispersed CdSe NBs. This also demonstrated that the prepared CdSe NBs were transferred perfectly without destroying their structure and morphology, which is beneficial to exert their intrinsic excellent optoelectronic properties after forming hybrid structure with CsPbBr_3_ MW.

**Figure 3:**
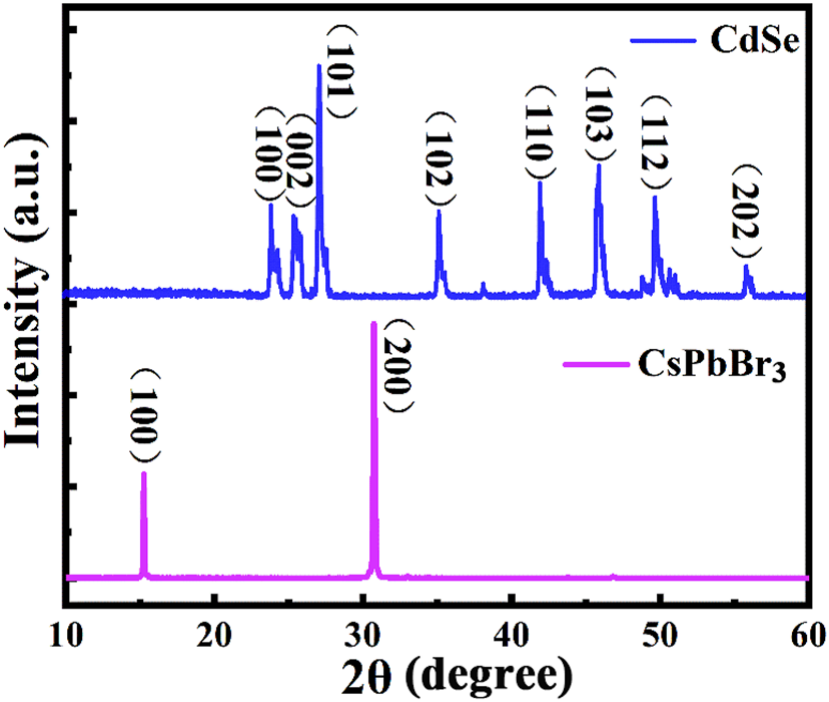
XRD of CdSe NBs (blue) and CsPbBr_3_ MWs (pink).

The crystal structures of CsPbBr_3_ MWs and CdSe NBs were further analyzed by X-ray diffraction (XRD), as shown in Figure 3. The blue diffraction peak in Figure 3 is the XRD data of CdSe NBs. The 2*θ* peaks located at 24.02°, 25.68°, 27.24°, 35.30°, 42.10°, 46.08°, 49.84°, and 55.98° correspond to (100), (002), (101), (102), (110), (103), (112), and (202) crystal planes, respectively, in good agreement with the standard card JCPDS NO:08–0459. The diffraction peaks in the figure are clear and without miscellaneous peaks, indicating that the prepared CdSe NBs have pure crystal phase and good crystallinity. There are two sharp and narrow characteristic peaks at 2*θ* = 15.2° and 2*θ* = 30.68° of CsPbBr_3_ MWs, indicating that the prepared MWs have good crystallinity, which is consistent with previous reports [35, 36]. In addition, the two characteristic peaks correspond to the (100) and (200) crystal planes of CsPbBr_3_, respectively, indicating that the typical layered structure of CsPbBr_3_ [37, 38].

Figure S5 of the Supporting Information shows UV–vis absorption spectra of CsPbBr_3_ MWs and CdSe NBs, respectively. It is obvious that CsPbBr_3_ MWs have strong absorption between 300–550 nm while CdSe NB has spectral absorption between 500–750 nm, which is helpful to fully utilize the sunlight and broad the response spectral range. According to Tauc equation: [39] (*α*h*ν*)^2^ = *C*(h*ν* − *E*_g_), where *α*, *hv*, *C,* and *E*_g_ represent absorption coefficient, light energy, proportionality constant, and optical band gap, respectively. By linear extrapolation, the band gaps of CsPbBr_3_ MWs and CdSe NBs are calculated to be 2.32 eV and 1.74 eV, respectively, which are consistent with those reported in previous literature [33, 40, 41].

**Figure 4:**
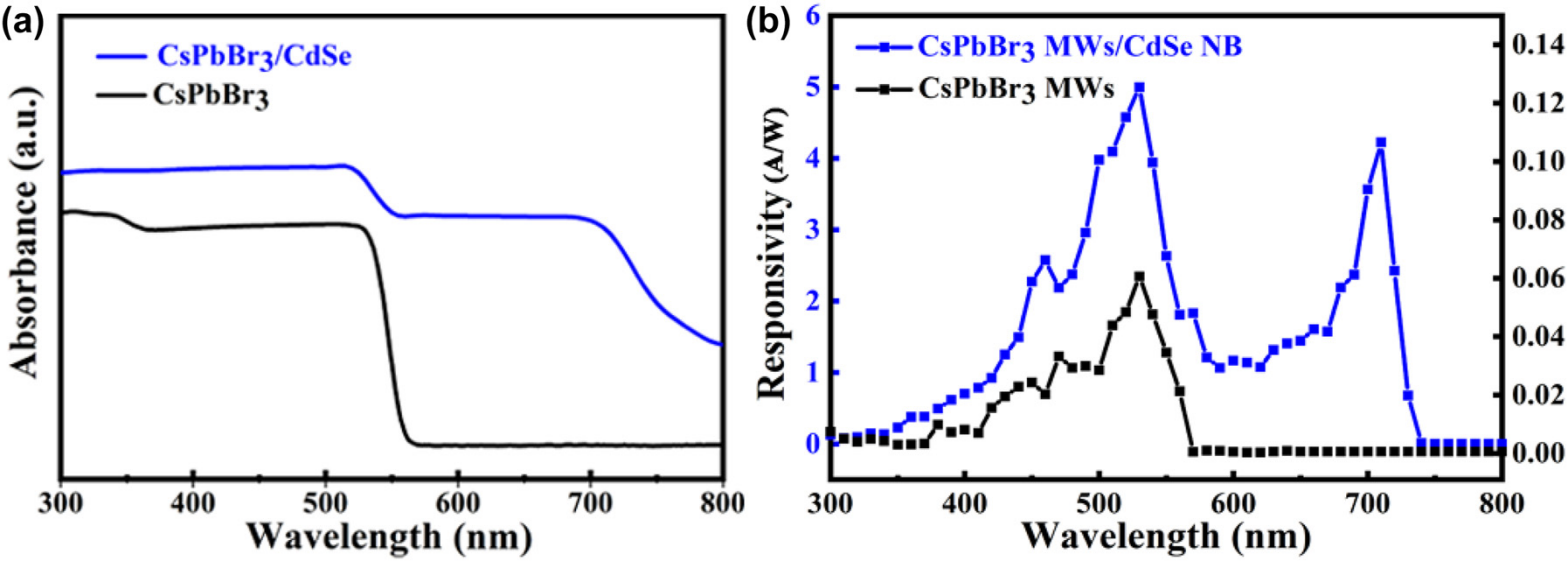
The spectral response of CdSe NB/CsPbBr_3_ MW hybrid structures. (a) UV–vis absorption spectra and (b) spectral responsivity of the CsPbBr_3_ MWs and CsPbBr_3_ MW/CdSe NB hybrid structure, respectively.

The spectral response of CdSe NB/CsPbBr_3_ MW hybrid structures was also investigated. Figure 4(a) shows UV–vis absorption spectra of CsPbBr_3_ MW and CdSe NB/CsPbBr_3_ MW hybrid structure in the range of 300–800 nm. Apparently, the hybrid structure exhibits enhanced absorption compared with the pure CsPbBr_3_ MWs in the ultraviolet to visible region, indicating its high light-harvesting capabilities. Two absorption onset values of the CdSe NB/CsPbBr_3_ MW hybrid structure are located at 530 nm and 710 nm, respectively. In contrast, the hybrid structures have enhanced absorption spectra not only at 300–550 nm but also at 550–800 nm, which can be regarded as the co-absorption of CdSe NB and CsPbBr_3_ MW. The enhanced light absorption is beneficial to the application of CdSe NB/CsPbBr_3_ MW hybrid devices. To better understand the optimal spectra of CsPbBr_3_ MW and CdSe NB/CsPbBr_3_ MW hybrid structure, the spectral response curves of CsPbBr_3_ MW and CdSe NB/CsPbBr_3_ MW hybrid structures were measured in the 300–800 nm range, respectively, as shown in Figure 4(b). It is clearly seen that CdSe NB/CsPbBr_3_ MW hybrid structure have increased photoresponsivity in the range of 300–740 nm and two photoresponsivity peaks appear at 530 nm and 710 nm, which is in good agreement with the results of UV–vis of the hybrid structure in Figure 4(a). Compared to single CsPbBr_3_, the photoresponsivity of the hybrid devices are improved by two orders of magnitude at the two peaks. Therefore, the photoelectric performance of the hybrid devices will be investigated systematically at 530 nm and 710 nm as representatives.

**Figure 5:**
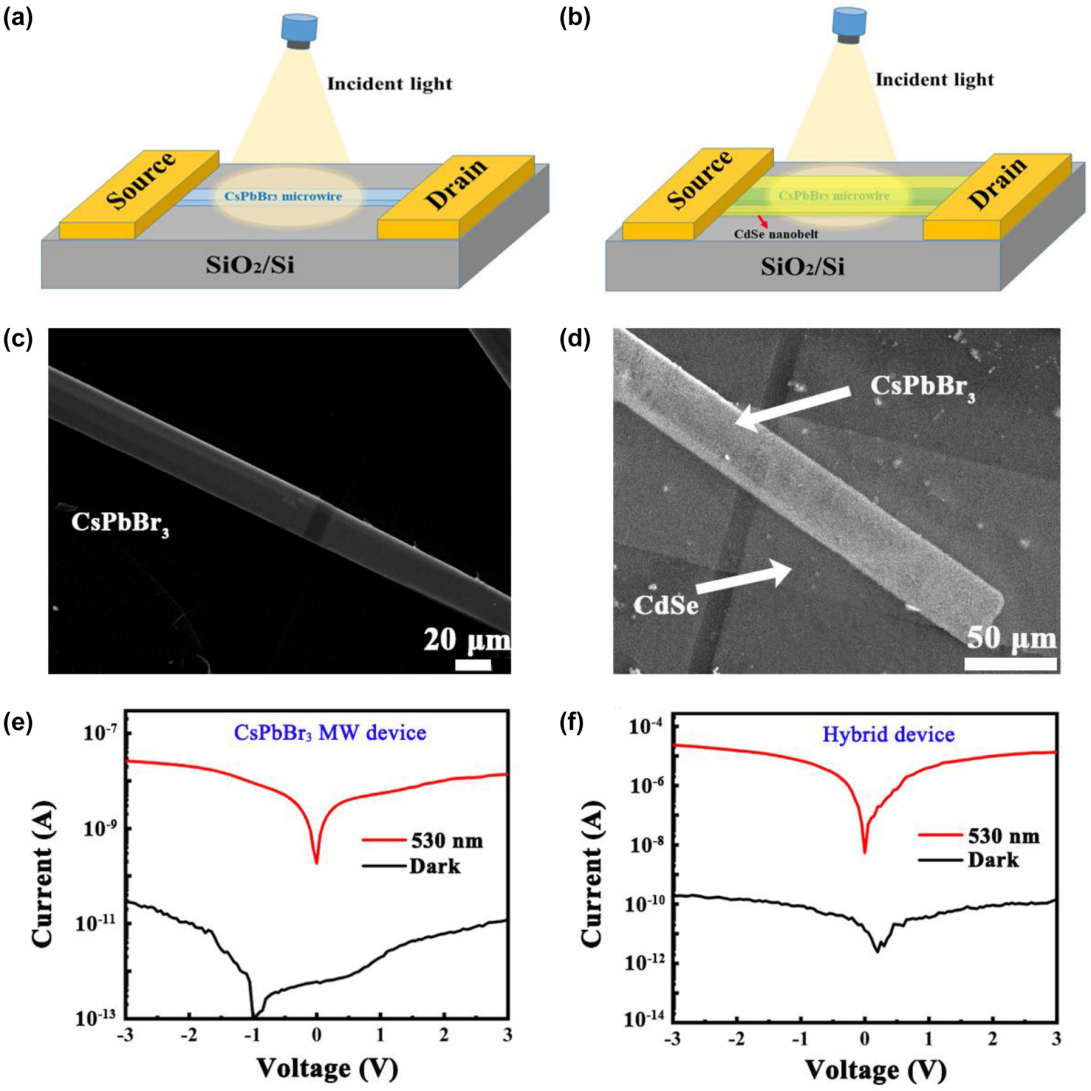
Schematic illustrations of (a) pure CsPbBr_3_ MW and (b) CdSe NB/CsPbBr_3_ MW hybrid structure devices. The SEM images of (c) pure CsPbBr_3_ MW and (d) CdSe NB/CsPbBr_3_ MW hybrid structure devices. *I*–*V* curves of (e) pure CsPbBr_3_ MW and (f) CsPbBr_3_ MW/CdSe NB devices under dark and 530 nm illumination.

The CdSe NB/CsPbBr_3_ MW 1D hybrid devices were later built and the optoelectronic characteristics were measured under dark and 530 nm illumination conditions. The optoelectronic characteristics of pure CsPbBr_3_ MW devices were also investigated for comparison. Figure 5(a)–(d) are the schematic illustrations and the corresponding SEM images of pure CsPbBr_3_ MW and CdSe NB/CsPbBr_3_ MW hybrid structure devices, respectively. From Figure 5(d), the profile of the CsPbBr_3_ MW underneath can be clearly seen from the CdSe NB side indicating that the CdSe NB is very thin, which can ensure a good light transmission and realize the co-absorption of light. Figure 5(e) and (f) plots the *I*–*V* curves of the pure CsPbBr_3_ MW and the CsPbBr_3_ MW/CdSe NB devices under dark and 530 nm illumination with the power density of 1.6 mW/cm^2^, where the effective areas of the devices were 4.5 × 10^−6^ cm^2^ and 8 × 10^−6^ cm^2^, respectively. At 3 V bias, the photocurrent and dark current of the CsPbBr_3_ MW/CdSe NB hybrid devices (pure CsPbBr_3_ MW devices) are 11.7 µA (13.7 nA) and 233 pA (12.4 pA), respectively, where the *I*_on_/*I*_off_ ratio of the hybrid device (5.02 × 10^4^) is improved by at least one order of magnitude than that of single CsPbBr_3_ MW photodetectors (1.107 × 10^3^). In comparison, it can be seen clearly that there is a pronounced zero-drift (∼1 eV) for the dark current of pure CsPbBr_3_ MW device, which arises from the mobile ion-drift induced current in CsPbBr_3_ [42, 43]. More interestingly, the zero-drift nearly disappeared as it combined with CdSe NB to form hybrid structure. The following theoretical calculations show that the phenomenon is attributed to the interface band gap states, which promotes the tunneling of electrons between CsPbBr_3_ and CdSe interface under dark condition and results in a slight increase in dark current from 12.4 to 233 pA. The increased dark current overwhelms the mobile ion-drift induced current in the hybrid structure and results in the zero-drift disappeared.

**Figure 6:**
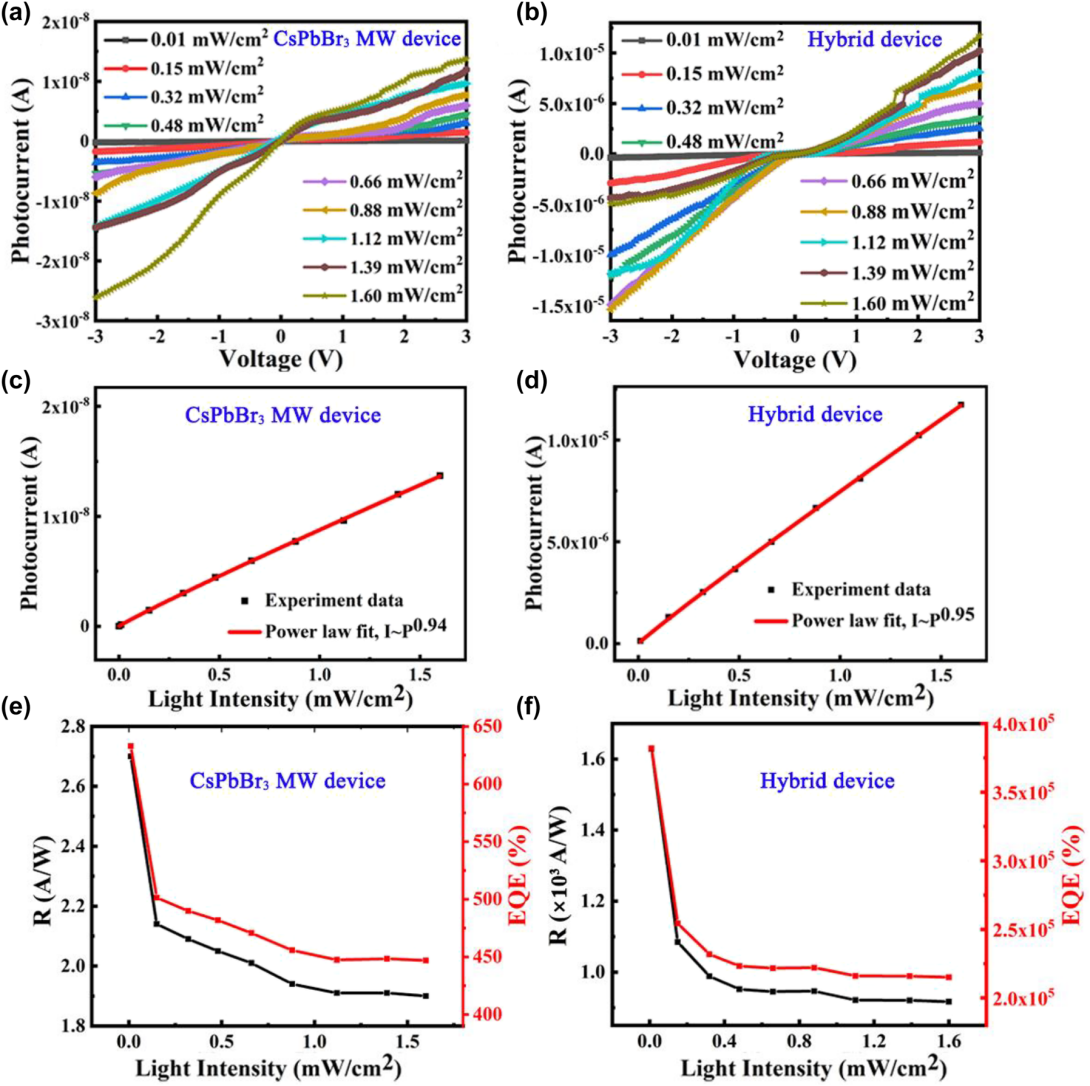
Performance of CdSe NB/CsPbBr_3_ MW photodetector. (a) and (b) The *I*–*V* curves, (c) and (d) the photocurrent, (e) and (f) the responsivity and EQE of pure CsPbBr_3_ MW and CdSe NB/CsPbBr_3_ MW hybrid devices with increasing light intensity under 530 nm laser and 3 V bias conditions.

Figure 6(a) and (b) shows the current–voltage (*I*–*V*) curves of CsPbBr_3_ MW and CdSe NB/CsPbBr_3_ MW devices tested at 530 nm irradiation with different power densities. It was found that the photocurrent increases with the increase of power density, indicating that the efficiency of photocarrier is proportional to the number of absorbed photons. The relation between current and optical power density of CsPbBr_3_ MW and CdSe NB/CsPbBr_3_ MW devices are plotted and fitted by the following formula: *I*_P_ = *αP* ^*θ*^, where *I*_P_, *α,* and *P* represent photocurrent, proportionality constant, and incident light intensity, respectively. The index *θ* determines the photocurrent response of the device to the light intensity, and *θ* = 1 indicates the ideal value that the photocurrent is proportional to the light intensity. The best *θ* fitting values of CsPbBr_3_ MWs and CdSe NB/CsPbBr_3_ MW devices are 0.94 and 0.95 (see Figure 6(c) and (d)), respectively, which are slightly less than the ideal value 1, indicating that CsPbBr_3_ MW and CdSe NB/CsPbBr_3_ MW devices have excellent optical switching ability and fewer defects.

In order to quantitatively evaluate the performance of CsPbBr_3_ MWs and CdSe NB/CsPbBr_3_ MW photodetectors, the responsivity (*R*_*λ*_), external quantum efficiency (EQE) and specific detectivity (D*) were calculated by the following formulas: [38, 44], [45], [46], [47] $R_{\lambda }=\frac{I_{\text{P}}-I_{\text{d}}}{P_{0}A}$ , EQE = *hcR*_*λ*_/*qλ* and $D^{*}=R_{\lambda }\sqrt{\frac{A}{2qI_{\text{d}}}}$ , where *I*_P_ and *I*_d_ are the photocurrent and dark current respectively, *P*_0_ is the power density of the incident light, *A* is the effective area of device, *h* is Planck’s constant, *c* is the speed of light, *q* is the fundamental charge, and *λ* is the wavelength of the incident light. According to the above formulas, the relationship between the *R*, EQE, and the optical power density of pure CsPbBr_3_ MW and CdSe NB/CsPbBr_3_ MW photodetectors under the 3 V bias of 530 nm illumination were calculated, respectively, as shown in Figure 6(e) and (f). The results show that the maximum values of *R*, EQE and D* of pure CsPbBr_3_ MW photodetector are 2.7 A/W, 632%, and 2.9 × 10^10^ Jones, respectively. By comparison, it was found that the maximum values of *R* (1.63 × 10^3^ A/W), EQE (3.8 × 10^5^%) and D* (5.33 × 10^12^ Jones) of CdSe NB/CsPbBr_3_ MW hybrid photodetector are all improved by at least two orders of magnitude compared to the single CsPbBr_3_ photodetector, respectively. In addition, the photoelectric performance of CdSe NB/CsPbBr_3_ MW hybrid photodetectors was tested at 710 nm illumination, as shown in Figure S6. The results show that the values of *θ*, *R,* and EQE of the hybrid device have the same magnitude compared with those tested at 530 nm illumination. Obviously, the photoelectric performance of the photodetector based on CdSe NB/CsPbBr_3_ MW hybrid structure dramatically increased compared to the single CsPbBr_3_ MW photodetector. It is observed that the *R* and EQE values decrease significantly with increasing light intensity, indicating the presence of non-negligible compound losses in the hybrid photodetector caused by interface band gap states.

**Figure 7:**
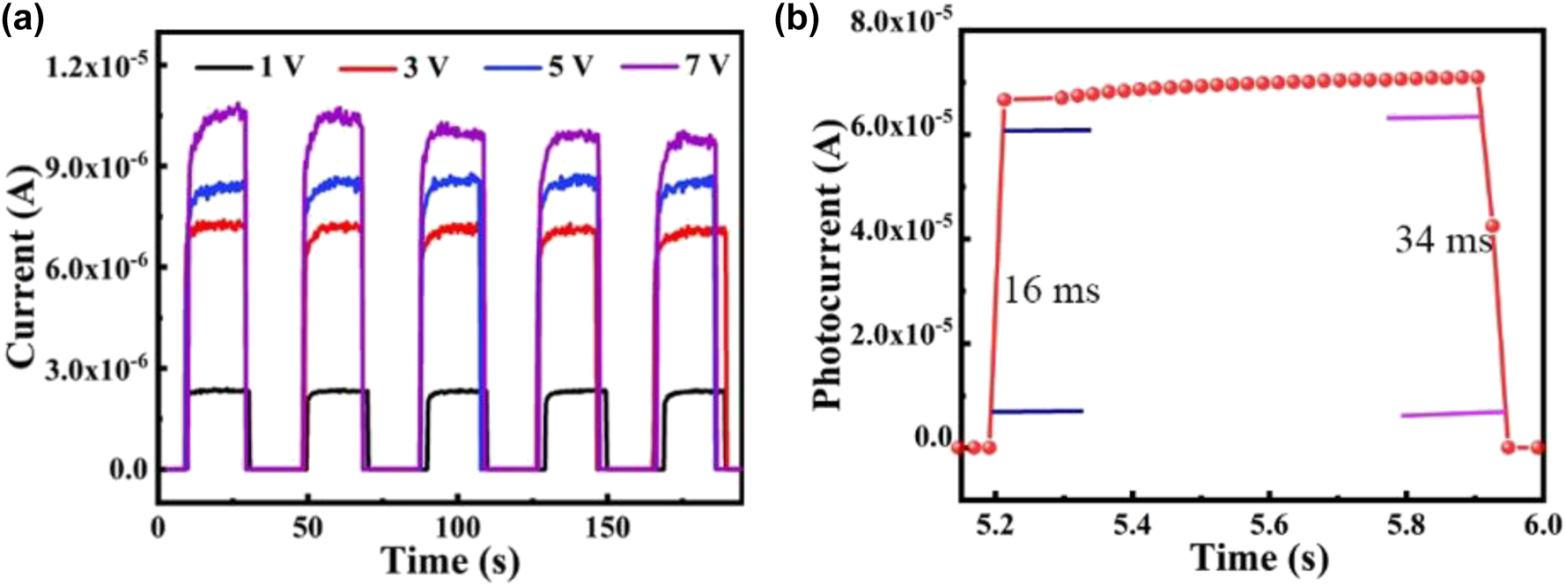
I−T characteristic curves of CdSe NB/CsPbBr_3_ MW photodetector. (a) Photocurrent versus time plot of CdSe NB/CsPbBr_3_ MW hybrid device under incident light of 530 nm with bias voltages of 1 V, 3 V, 5 V, and 7 V, respectively. (b) Rising and falling edges in a single cycle at a bias voltage of 3 V.

Figure 7(a) shows the *I*–*T* curves measured by continuous on-off at bias voltages of 1, 3, 5, and 7 V under 530 nm illumination. It can be seen that the current of the CdSe NB/CsPbBr_3_ MW device gradually increases with the increase of bias voltage. The time-resolved current change under periodic illumination clearly shows the repeatable and reversible optical sensing behavior of CdSe NB/CsPbBr_3_ MW hybrid material. Response speed is one of the most important parameters to describe photodetectors. Figure 7(b) shows the rise and the decay edge of the device tested by an oscilloscope, from which the rise time (from 10% to 90% of the maximum current) and decay time (from 90% to 10% of the maximum current) were calculated to be 16 ms and 34 ms, respectively. The values have slightly increased relative to those of the pure CsPbBr_3_ MW device (10/8.3 ms, see Figure S7) but still close to the same order of magnitude, indicating the photodetector still keep a good on/off switching performance.

For better comparison, the reported optoelectronic properties of CsPbBr_3_-based optoelectronic devices are summarized, as shown in Table 1. The optoelectronic performance of fabricated pure CsPbBr_3_ MW photodetector is comparable with that of reported literatures [48]. When the CsPbBr_3_ MW hybridized with CdSe NB, the optoelectronic performance of the hybrid device is significantly improved, and most key parameters of the hybrid device were improved by at least one order of magnitude compared with those of the single CsPbBr_3_ MW device. Meanwhile, the CdSe NB/CsPbBr_3_ MW hybrid devices is competitive compared with most other CsPbBr_3_-based hybrid devices because of the better optoelectronic performance as well as the wider spectral response range from 300 to 740 nm.

**Table 1:** Comparison of performance parameters of CsPbBr_3_-based photodetector device.

Device structure	Bias voltage [V]	EQE [%]	*R* [A/W]	*D* ^*^ [Jones]	*I*_P_/*I*_d_ ratio	Rise/decay time [ms]	Ref.
CsPbBr_3_/ZnO	10	–	4.25	–	1.1 × 10^4^	0.21/0.24	[26]
CsPbBr_3_/CNT	10	7488	31.1	–	–	0.016/0.38	[27]
MoS_2_/CsPbBr_3_	10	302	4.4	2.5 × 10^10^	10^4^	0.72/1.01	[25]
CsPbBr_3_/Graphene	0.2	6 × 10^6^	2 × 10^4^	8.6 × 10^10^	–	3.1 × 10^3^/2.42 × 10^4^	[28]
CsPbBr_3_/WS_2_	2	1.58 × 10^4^	57.2	1.36 × 10^14^	10^9.83^	2/2	[24]
CsPbBr_3_/GaN	10	420	1.78	6.5 × 10^13^	<100	1.35 × 10^4^/2.7 × 10^3^	[23]
CsPbBr_3_/PbS	5	–	15	2.65 × 10^11^	–	102/96	[49]
CsPbBr_3_ MWs	3	7540	20	9.38 × 10^10^	5.38 × 10^3^	0.25/0.29	[48]
CsPbBr_3_ MW	3	632	2.7	2.9 × 10^10^	1.11 × 10^3^	10/8.3	This work
CsPbBr_3_ MWs/CdSe	3	3.8 × 10^5^	1.63 × 10^3^	5.33 × 10^12^	5.02 × 10^4^	16/34	This work

**Figure 8:**
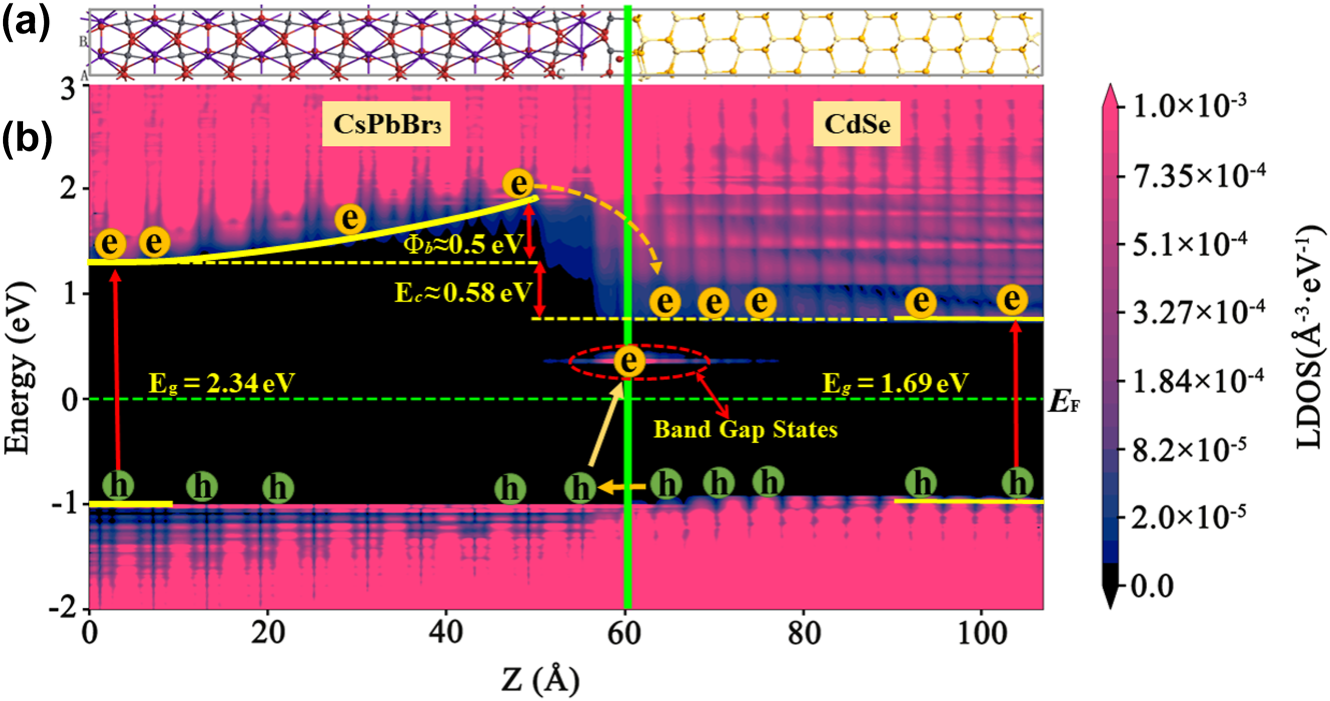
Crystal structure and band arrangement of CsPbBr_3_/CdSe heterostructure. (a) Structure and cell used in the calculation of the CsPbBr_3_/CdSe heterostructure. (b) Band diagram showing the spatial resolved PLDOS across the CsPbBr_3_/CdSe heterostructure.

In order to reveal the physical mechanism of the improved optoelectronic performance, the band alignment at the interface between CsPbBr_3_ and CdSe were investigated based on the first-principles atomistic simulations. To construct more realistic models of the interface, the two-probe device model [50] at equilibrium (i.e., zero-bias voltage) was used to describe the infinite, nonperiodic interface. Figure 8 shows the calculated projected local density of states (PLDOS) of the CsPbBr_3_/CdSe heterostructure by using QuantumATK package [51]. The calculated band gaps of CsPbBr_3_ and CdSe are 2.34 eV and 1.69 eV, respectively, which are in good agreement with the experimental results. There are two band alignment parameters in heterostructure, one is the interface potential barrier (*Φ*_b_), which is defined as the distance between the CsPbBr_3_ conduction band minimum (CBM) at the interface and in its bulk region. Another band alignment parameter is the conduction (valence) band offset Δ*E*_c_ (Δ*E*_v_) that we define as the distance between the CBM (valence-band maximum, VBM) in the bulk CsPbBr_3_ region and that of the CdSe region. It can be seen that there is a large *Φ*_b_, which can suppress the dark current in the CsPbBr_3_/CdSe heterostructure in dark condition. The large band offset (Δ*E*_c_) promoted the separation and transfer of photoexcited electrons and holes in heterostructure. Under light illumination, a larger number of the photoexcited electrons accumulated in the interface region and lowers the effective barrier height, which makes the electron transfer from CB of CsPbBr_3_ to CB of CdSe more easily. In addition, the valence band offset Δ*E*_v_ is quite small, which lead to the holes can transfer from VB of CdSe to VB of CsPbBr_3_ more easily, resulting in the effective separation of electrons and holes. Besides, there is a band gap states at the interface, which arises from the interfacial unsaturated Br and Se atoms by further theoretical calculations. The band gap states also promote the transition and tunneling of some electrons at the interface from VBM of CsPbBr_3_ to CBM of CdSe, resulting in the increased photocurrent compared with the isolated CsPbBr_3_ MW device. It should be noted that the interface band gap states also have an effect on the dark current. It was found that the dark current of the CsPbBr_3_/CdSe hybrid device was slightly increased compared to the single CsPbBr_3_ MW device but still remains a small value. Obviously, the promotion effect of interface band gap states on dark current is greater than the inhibition effect of interface potential barrier *Φ*_*b*_ on dark current in our hybrid device.

## 4 Conclusions

The high quality 1D CdSe NB/CsPbBr_3_ MW hybrid structure photodetectors are successfully fabricated. Compared with the pure CsPbBr_3_ MW photodetector, the spectral response range of CdSe NB/CsPbBr_3_ MW hybrid photodetector is significantly broadened from the original 300–570 nm to 300–740 nm. More importantly, the performances of CdSe NB/CsPbBr_3_ MW hybrid device have been significantly improved, with *I*_on_/*I*_off_ ratio, *R*, EQE and D* values up to 5.02 × 10^4^, 1.63 × 10^3^ A/W, 3.8 × 10^5^%, and 5.33 × 10^12^ Jones, respectively. The improved performance of CdSe NB/CsPbBr_3_ MW hybrid photodetector is attributed to three key aspects: (1) the excellent intrinsic optoelectronic properties of CsPbBr_3_ and CdSe as well as their unique 1D morphology, (2) the prepared 1D CdSe NB/CsPbBr_3_ MW hybrid structures have fewer grain boundaries and defects because of their high crystalline quality, which is conducive to the separation and transport of photogenerated carriers, and (3) the perfect band alignment between CdSe and CsPbBr_3_ forms of a unique charge transfer mechanism, in which CdSe acts as an electron transport layer. This work provides an approach for building high-performance detectors.

## Supplementary Material

Supplementary Material Details
