# Supplementary material for: Constructing high-quality 1D nano/microwire hybrid structure for high-performance photodetectors based on CdSe nanobelt/perovskite microwire
Source: Nanophotonics. 2023 Feb 28;12(7):1347–57. doi: 10.1515/nanoph-2023-0106 (PMC11635968; doi:10.1515/nanoph-2023-0106)
Supplement: Supplementary file 1 — Supplementary Material Details [file j_nanoph-2023-0106_suppl.docx]

**Supporting Information**

Li Ren, Qiuhong Tan^[[1]](#footnote-1)^⁎, Kunpeng Gao, Peizhi Yang, Qianjin Wang* and Yingkai Liu

**Constructing High-Quality 1D Nano/Microwire Hybrid Structure for High-Performance Photodetectors Based on CdSe Nanobelt/Perovskite Microwire**


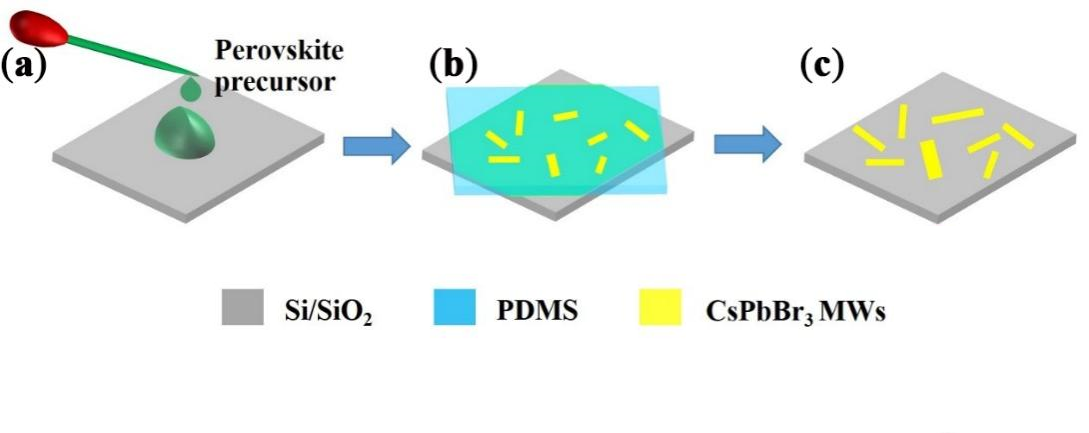


**Figure S1:** Flow chart of preparation of CsPbBr_3_ microwire.


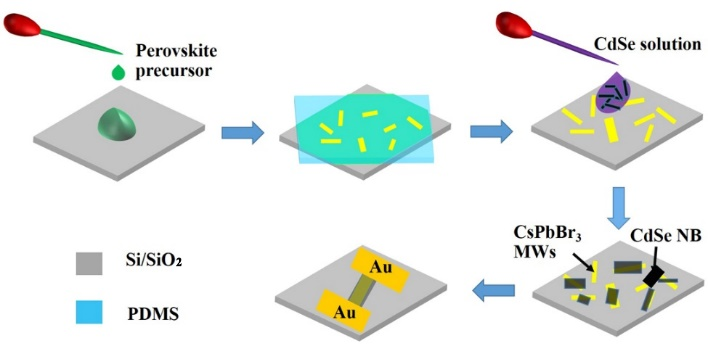


**Figure S2:** Flow chart of preparation of CdSe NB/CsPbBr_3_ MW hybrid structure photodetector.


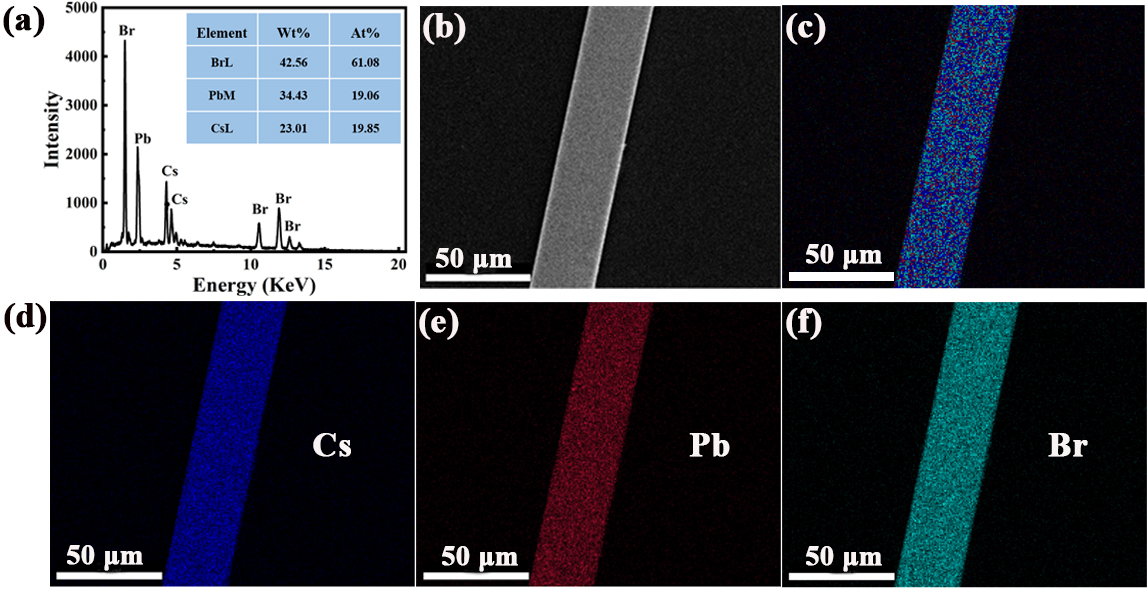


**Figure S3:** (a) EDS elemental analysis. (b-f) Enlarged SEM image of single CsPbBr_3_ MW and corresponding EDS element mapping diagram of Cs, Pb and Br elements, respectively.


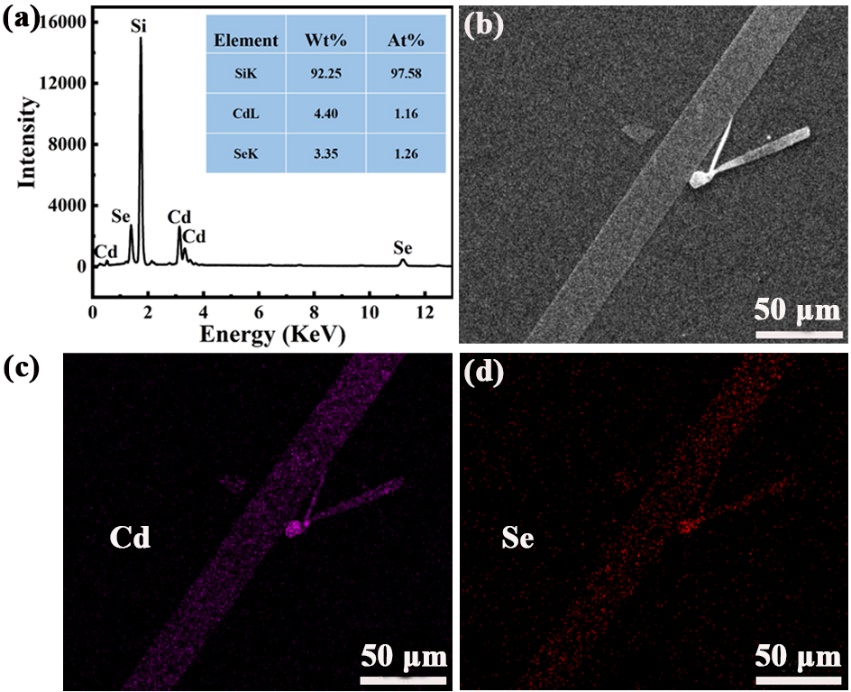


**Figure S4:** (a) EDS elemental analysis. (b-d) Enlarged SEM image of single CdSe NB and corresponding EDS element mapping diagram of Cd and Se elements, respectively.


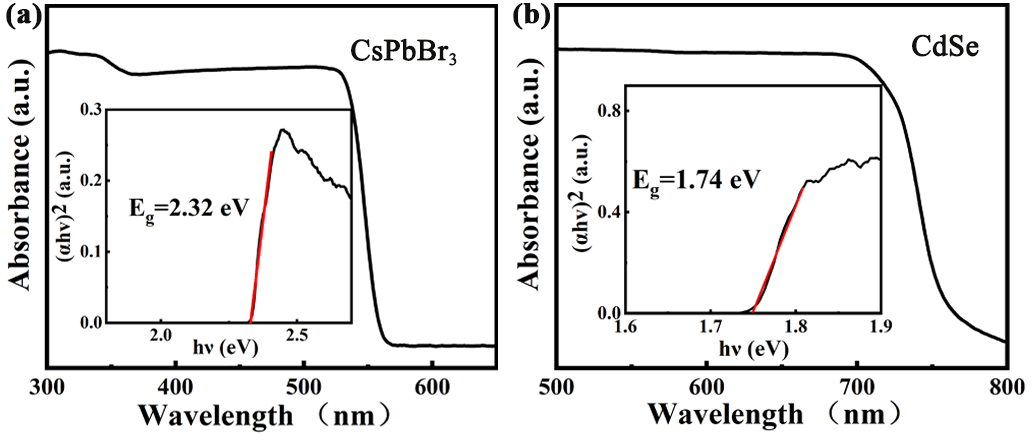


**Figure S5:** UV–vis absorption spectra of (a) the CsPbBr_3_ MWs and (b) CdSe NB, respectively; Inserts are the corresponding relationship between (*αhv*)^2^ and photon energy.

**
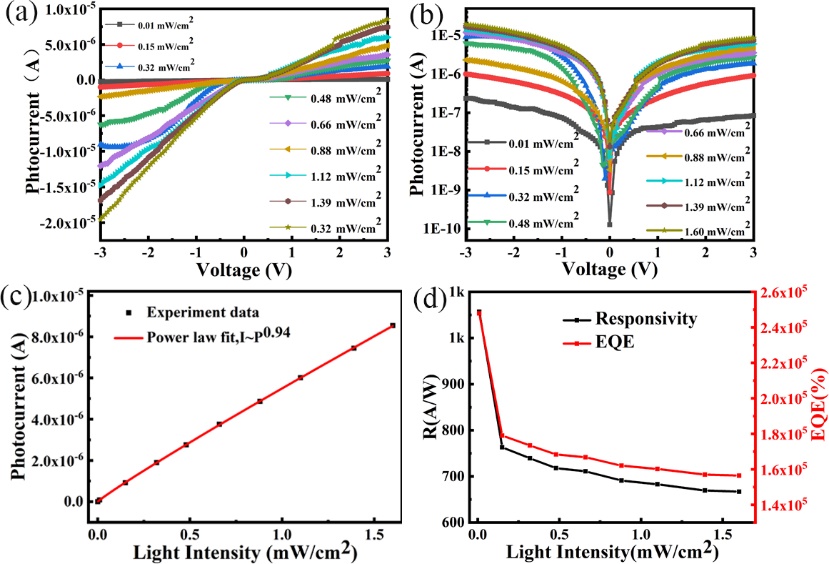
**

**Figure S6:** (a-b) The I-V curve and its logarithmic I-V curve, (c) the relationship between photocurrent and optical density, and (d) the relationship between responsivity and optical power density of CdSe NB/CsPbBr_3_ MW devices under 710 nm laser and 3 V bias conditions.


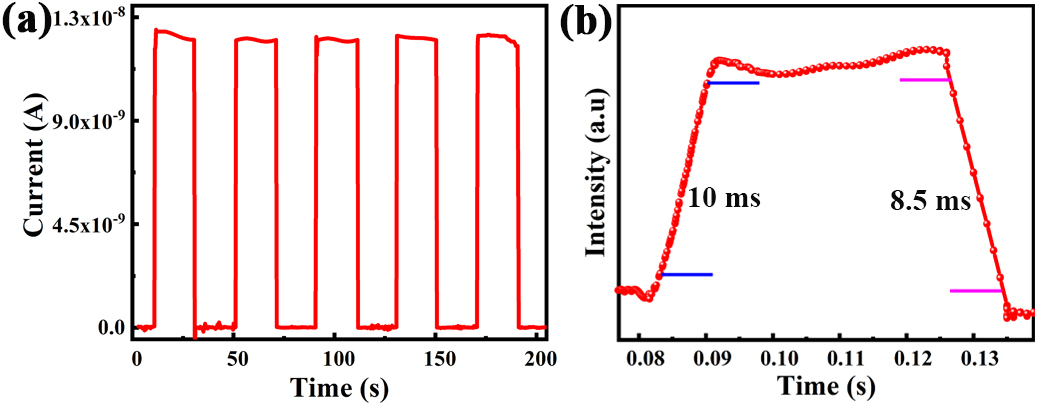


**Figure S7:** CsPbBr_3_ MW device with incident light of 530 nm and bias voltages of 3 V. (a) Plot of current versus time for a cyclically switched light source; (b) Rising and falling edges in a single cycle.

1. **^*^Corresponding authors:** **Qiuhong Tan and** **Qianjin Wang,** College of Physics and Electronic Information, Yunnan Normal University, Yunnan Kunming 650500, China; and Yunnan Provincial Key Laboratory for Photoelectric Information Technology, Yunnan Normal University, Yunnan Kunming 650500, China; and Key Laboratory of Advanced Technique & Preparation for Renewable Energy Materials, Ministry of Education, Yunnan Normal University, Kunming 650500, China.

   E-mail: [tanqiuhong1@126.com](mailto:tanqiuhong1@126.com) (Q. Tan), [qjwang@xtu.edu.cn](#mailto:qjwang@xtu.edu.cn) (Q. Wang).

   <https://orcid.org/0000-0003-1474-0918> (Q. Wang).

   **Li Ren and Kunpeng Gao,** College of Physics and Electronic Information, Yunnan Normal University, Yunnan Kunming 650500, China.

   **Peizhi Yang,** Key Laboratory of Advanced Technique & Preparation for Renewable Energy Materials, Ministry of Education, Yunnan Normal University, Kunming 650500, China.

   **Yingkai Liu,** College of Physics and Electronic Information, Yunnan Normal University, Yunnan Kunming 650500, China; and Yunnan Provincial Key Laboratory for Photoelectric Information Technology, Yunnan Normal University, Yunnan Kunming 650500, China; and Key Laboratory of Advanced Technique & Preparation for Renewable Energy Materials, Ministry of Education, Yunnan Normal University, Kunming 650500, China. https://orcid.org/0000-0003-0041-1793 [↑](#footnote-ref-1)
